# Cholesterol Dependence of Collagen and Echovirus 1 Trafficking along the Novel α2β1 Integrin Internalization Pathway

**DOI:** 10.1371/journal.pone.0055465

**Published:** 2013-02-05

**Authors:** Elina Siljamäki, Nina Rintanen, Maija Kirsi, Paula Upla, Wei Wang, Mikko Karjalainen, Elina Ikonen, Varpu Marjomäki

**Affiliations:** 1 Department of Biological and Environmental Science/Nanoscience Center, University of Jyväskylä, Jyväskylä, Finland; 2 Institute of Biomedicine, University of Helsinki, Helsinki, Finland; 3 Minerva Foundation Institute for Medical Research, Helsinki, Finland; University of Bergen, Norway

## Abstract

We have previously shown that soluble collagen and a human pathogen, echovirus 1 (EV1) cluster α2β1 integrin on the plasma membrane and cause their internalization into cytoplasmic endosomes. Here we show that cholesterol plays a major role not only in the uptake of α2β1 integrin and its ligands but also in the formation of α2 integrin-specific multivesicular bodies (α2-MVBs) and virus infection. EV1 infection and α2β1 integrin internalization were totally halted by low amounts of the cholesterol-aggregating drugs filipin or nystatin. Inhibition of cholesterol synthesis and accumulation of lanosterol after ketoconazole treatment inhibited uptake of collagen, virus and clustered integrin, and prevented formation of multivesicular bodies and virus infection. Loading of lipid starved cells with cholesterol increased infection to some extent but could not completely restore EV1 infection to control levels. Cold Triton X-100 treatment did not solubilize the α2-MVBs suggesting, together with cholesterol labeling, that the cytoplasmic endosomes were enriched in detergent-resistant lipids in contrast to αV integrin labeled control endosomes in the clathrin pathway. Cholesterol aggregation leading to increased ion permeability caused a significant reduction in EV1 uncoating in endosomes as judged by sucrose gradient centrifugation and by neutral red-based uncoating assay. In contrast, the replication step was not dependent on cholesterol in contrast to the reports on several other viruses. In conclusion, our results showed that the integrin internalization pathway is dependent on cholesterol for uptake of collagen, EV1 and integrin, for maturation of endosomal structures and for promoting EV1 uncoating. The results thus provide novel information for developing anti-viral strategies and more insight into collagen and integrin trafficking.

## Introduction

The plasma membrane is a complex organelle composed of various glycerophospholipids, sphingolipids, cholesterol and a diverse set of proteins. The knowledge of plasma membrane organization has evolved from the first, homogenous fluid-mosaic model to the present model of membrane microdomains, each having their own protein and lipid composition [1]. Lipid rafts are small, dynamic microdomains enriched in sphingolipids, cholesterol and associated proteins, such as glycosylphosphatidylinisotol (GPI)-anchored proteins [2]. They have significant functions for example in sorting, signaling and endocytosis, and their dysfunction has been linked to pathological states [3]–[5]. Many pathogens, including bacteria, parasites and viruses, have been found to hijack lipid rafts during cell entry [6].

We have recently studied the cell entry pathway of a human picornavirus, echovirus 1 (EV1), as a model system to understand events that occur during integrin-dependent cargo uptake [7]–[10]. Our biochemical and imaging studies have shown that EV1 is internalized in complex with its receptor, α2β1 integrin into tubulovesicular structures, which then gradually mature into α2 integrin-specific multivesicular bodies (α2-MVBs) [9], [11]. The early entry is independent of clathrin and caveolin-1 but is instead regulated by a set of typical macropinocytic regulators, namely PKCα [7], [10], PLC, Rac1, Pak1 [11] and CtBP1/BARS [12]. This pathway differs from typical integrin recycling as virus-induced clustering directs α2β1 integrin into a non-recycling pathway [13], [14]. Targeting of α2β1 integrin into the α2-MVBs drives enhanced turnover of integrin, which is blocked by inhibition of neutral calpain proteases [14]. Recently, we found out that soluble collagen, the physiological ligand for α2β1 integrin, also clusters integrin and is co-internalized to a non-lysosomal pathway, which is sensitive to calpain inhibition [14]. It thus seems probable that EV1 has learned to use this non-recycling collagen uptake pathway for its own benefit.

α2β1 integrin cofractionates with detergent-resistant membranes [10]. On the plasma membrane, α2β1 integrin colocalizes first with GPI-anchored proteins but is sorted out from GPI-anchored proteins during internalization [10]. However, later, the multivesicular bodies become increasingly positive for caveolin-1 suggesting that stable lipid microdomains exist in these endosomes. Interestingly, our previous intraendosomal pH measurements have shown that α2-MVBs are not acidic and do not associate with lysosomal structures [14], [15]. As these structures have proven to be important for viral uncoating and genome egress for replication, as well as for location of enhanced integrin turnover, the characterization and role of the lipid microdomains is important to understand the structure and function of these novel non-acidic multivesicular bodies.

Many viruses, such as HIV [16], hepatitis C virus [17], [18], West Nile virus [19], vaccinia virus [20] and poliovirus [21] depend on plasma membrane cholesterol for efficient cell entry and replication. However, the putative role of these lipids in internalized endosomes has not been demonstrated. The cellular cholesterol concentration varies largely depending on the compartment membrane. Most of the cholesterol can be found at the plasma membrane, but also many intracellular organelles contain raft-like microdomains [22]–[28]. In addition, studies on acidic late endosomes have revealed that raft-like membranes - rich in cholesterol, sphingomyelin and raft proteins - can be found not only on the limiting membranes but also on the internal membranes of these multivesicular organelles [25]. In late endosomes, cholesterol accumulation can lead to disturbed vesicle trafficking from the structures [29], underlining the importance of membrane cholesterol in these intracellular organelles.

In this study, we investigated whether cholesterol plays a role in ligand uptake, virus uncoating in endosomes and the biogenesis of the non-acidic multivesicular bodies. We showed that the formation of α2-MVBs is highly dependent on cholesterol and that perturbation of cholesterol inhibits collagen and EV1 entry as well as virus uncoating and infection.

## Materials and Methods

### Cells, Viruses, Antibodies and Reagents

Experiments were performed using a human osteosarcoma cell line, overexpressing the α2 integrin subunit (SAOS-α2β1 cells, clone 45) that were originally published by Ivaska et al. [30]. In addition, A549 cells (ATTC) and MDA-MB-231 cells (from Dr. Johanna Ivaska, Turku, Finland) were used. EV1 (Farouk strain, ATCC) was propagated in African green monkey kidney (GMK, ATTC) cells and purified in sucrose gradients as described previously [9]. Monoclonal antibodies against α2 integrin subunit (A211E10, a gift from Dr. Fedor Berditchevski, Birmingham, UK), αV integrin subunit (L230, ATCC), Lysosomal-associated membrane protein 1 (Lamp-1) (Santa Cruz Biotechnology) and dsRNA (J2, English & Scientific Consulting Bt.) and polyconal rabbit antisera against EV1 [9], rabbit anti-collagen type I (Cederlane Laboratories), rabbit anti-mouse IgG (Sigma) and goat anti-rabbit or anti-mouse IgG conjugated with Alexa 488 or Alexa 555 (Invitrogen) were used. Nystatin,filipin III, ketoconazole, Fumonisin B_1_, methyl-β-cyclodextrin, cholesterol, octyl-β-D-glucopyranoside, 3-β-[2-(diethylamino)ethoxy]androst-5-en-17-one (U18666A) and poly-L-lysine were all purchased from Sigma, streptavidin Alexa 488 and FluoSpheres® carboxylate-modified microspheres, 0.02 µm yellow-green (505/515) from Invitrogen, Neutral red from Merck, [^35^S]methionine from Perkin Elmer, LX-112 from Ladd Research, protein-A-gold (CMC) and Vitrogen 100 from Angiotech Biomaterials.

### Thin Layer Chromatography

Lipids were extracted from SAOS-α2β1 cells as described in [31]. To separate cholesterol and its sterol precursors, lipids corresponding to the same protein amount were subjected to Ag-HPTLC [32] in chloroform:aceton (19∶1,v/v) as the solvent system. Lipid bands were visualized by charring.

### Preparation of Lipoprotein-deficient Serum (LPDS)

LPDS was prepared as described in [33].

### Internalization Experiment

In EV1 infectivity assays, EV1 was bound on cells for 1 h on ice and unbound virus was removed by washing cells 3 times with 0.5% BSA-PBS. Infection was allowed to proceed at +37°C for 6 to 7 h before 4% PFA-PBS fixation for 20–30 min. Integrin clustering with antibodies was done as previously described [10]. Briefly, α2 integrin antibody in 1% DMEM was bound for 45 min on ice. After washes with 0.5% BSA-PBS for 3 times, secondary clustering antibody was bound for 30 min and washed similarly. To measure the internalization of α2β1 integrin after drug treatments, cells were first preincubated various times with drugs at +37°C. After this, α2β1 integrin was clustered and let to internalize at +37°C for 2 h in the presence of the drugs. Cells were fixed with 3% PFA for 10 min at RT. To study the effect of Triton X-100 or filipin treatments on α2 or αV integrin containing structures, integrins were internalized for 2 h at +37°C. Then cells were treated with 0.2% Triton X-100 or filipin in 1% DMEM for 30 min at +4°C before fixation with 4% PFA-PBS for 20–30 min. Fluospheres were cointernalized with antibody clustered α2 integrin for 1 h (15 min pulse 1 µg/ml and 45 min chase) after which filipin was added for 2 h.

### Drug Treatments

Prior to the drug treatments coverslips were coated with poly-L-lysine (1 µg/ml) for 2 h at RT or o/n at +4°C to prevent detachment of the cells. Collagen coating was done by adding cold solution of 5 mg/ml collagen (Vitrogen 100) in PBS on coverslips on ice and incubated at +4°C o/n. To test the effects of cholesterol sequestering drugs on EV1 infection or internalization, nystatin (50 µg/ml) or filipin (1 µg/ml) was added onto cells in 1% DMEM either 30 min prior to virus addition or various times of post infection (p.i.). Cells were pretreated with 10 µM ketoconazole in 5% LPDS DMEM for 3 days, with 20 µg/ml Fumonisin B_1_ in 10% DMEM for 2 days or with 3 µg/ml U18666A o/n. All drugs were also present in internalization medium. To load cells with cholesterol, cells were starved in 5% LPDS DMEM for 24 h after which methyl-β-cyclodextrin (mβCD)-cholesterol complex (stock 300 mM and 50 mM, respectively) was added on cells for 0.5 h prior to EV1 binding on ice. 24 µM, 480 µM or 960 µM cholesterol concentrations were used.

### Immunofluorescence and Confocal Microscopy

Immunofluorescence labelings were done as described in [11], except the filipin staining was performed as in [34]. Shortly 4% PFA fixed cells were permebilized with 0.2% Triton X-100 in PBS for 5 min, after which they were treated with antibodies diluted in 3% BSA-PBS. In the case of filipin staining, cells were treated with 0.5 mg/ml filipin in PBS for 30 min at +37°C. Filipin permeabilized cells were then washed 2 times with PBS and labeled with antibodies diluted in 3% BSA-PBS. Finally cells were mounted in mowiol. Differential labeling of the surface and intracellular integrins were done as described previously [10]. Briefly, α2β1 integrin was first clustered and then allowed to internalize in the presence of anti-mouse Alexa 488 conjugate for 2 h. The cells were fixed with 3% PFA for 10 or 15 min in RT and labeled without permeabilization by using the anti-mouse Alexa 555 conjugate. Thus, plasma membrane-associated integrin was stained with both Alexa 488 and 555 conjugates, whereas green signal alone represented the internalized integrin clusteres. As an exception, ketoconazole internalization assay was done with biotinylated α2 integrin antibody, clustered with anti-mouse Alexa 555 (red) and surface bound integrin was labeled after fixation with strepavidin Alexa 488 (green). Samples were imaged with an Olympus microscope IX81 with Fluorview-1000 confocal setup or Zeiss Axiovert 100 M SP epifluorescence microscope with LSM510 confocal setup.

### Analysis of EV1 Uncoating by Sucrose Gradient Sedimentation and Neutral-red Labeled EV1 Photosensitivity Assay

Sucrose sedimentation assay was done as in [9]. [^35^S]methionine-labeled EV1 (40.000 cpm) was used and unbound virus was washed 3 times with 0.5% BSA-PBS. Filipin (1 µg/ml) and nystatin (50 µg/ml) were added after 15 minutes of incubation, and the infection was let to proceed up to 4 h. Cells were lysed with 100 mM octyl-β-D-glucopyranoside for 30 min on ice. The supernatant was layered on a 5 to 20% (WT/vol) sucrose gradient and centrifuged for 2 h at 4°C and 35 000 rpm in Beckman SW41Ti rotor. Fractions (500 µl) were analysed for radioactivity with a scintillation counter.

The neutral-red labeled EV1 (NR-EV1) was produced in GMK cells that were infected in the presence of 10 µg/ml NR. The virus was released at 20 h p.i. by freeze-thawing the cells three times and harvested by centrifugation and saving the supernatant. All steps were performed in dark. NR-EV1 was used without further purification. NR-EV1 infection was done as described above and at indicated times p.i., the cells were exposed to white light for 10 min at room temperature and subsequently transferred to +37°C. The control cells were not exposed to light.

### Quantification of 3D Data Using BioImageXD

To analyse the internalized α2 or αV integrin-positive structures, free, open source software package BioImageXD was used [35] In order to quantify the intensities, numbers of objects and the volumes of internalized integrin structures, the data was subjected to thresholding, connected component labeling, and structures smaller than 3 voxels were excluded from further processing. To quantify the level of internalization, 30 cells from three independent experiments were randomly selected and optically sectioned using confocal microscope. Colocalization was evaluated from the center slice of the cell and the colocalization thresholds were set manually in order to prevent background fluorescence and fluorescence from diffuse staining to affect the measurement. Calculations of the ratio of surface versus internalized integrin were done with the internalization algorithm embedded in BioImageXD as described previously [11].

### Electron Microscopy

Samples were prepared as in [11]. Briefly, α2 integrin antibody (A211E10) was bound to the cells for 1 h on ice, followed by treatments with rabbit anti-mouse and 10 nm protein A -gold (G Posthuma, and J Slot, University Medical Centre Utrecht, The Netherlands), both for 1 h on ice. α2β1 integrin were then allowed to internalize at +37°C for various time periods (30 min to 3.5 h) The cells were fixed in 2.5% glutaraldehyde in 0.1 M phosphate buffer (pH 7.4) for 1 h, postfixed with 1% osmium tetroxide for 1 h in the same buffer, dehydrated in ethanol, stained with uranyl acetate, and embedded with LX-112.

### Statistical Testing

For statistical comparison of results one-tail *t*-test was used. For results announced as percentages or ratios, arcsin√ transformation was applied first to convert results to follow normal distribution. When testing mean values in EV1 infection assay+filipin or nystatin binomial *t*-test was used.

## Results

### Cholesterol is Needed for α2β1 Integrin Internalization

**Figure 1:**
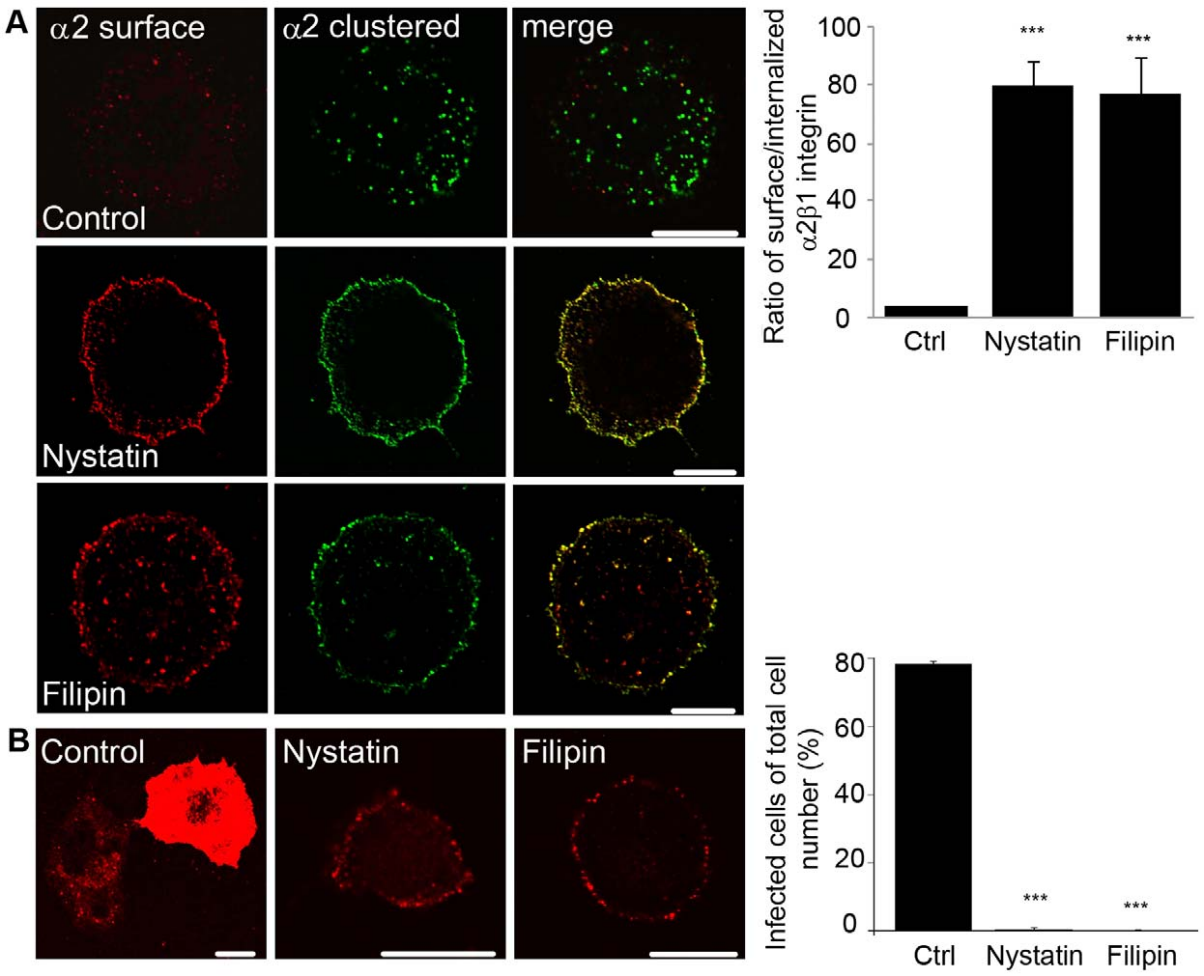
Cholesterol sequestering drugs inhibit α2β1 integrin internalization and EV1 infection. A) Differential staining of the internalized (green) and surface-bound (red or yellow) α2β1 integrin 2 h after internalization. Nystatin (50 µg/ml), filipin (1 µg/ml) or equal amount of DMSO (control) were used. Bars, 10 µm. The ratio of surface versus internalized α2β1 integrin was determined using the internalization algorithm in BioImageXD software and the higher ratio means lower amount of internalization. Results are expressed as mean values measured from 30 cells from 3 independent experiments ± standard error (SE). ***P<0.001. B) The effect of nystatin (50 µg/ml) or filipin (1 µg/ml) on EV1 infectivity was determined. Results are expressed as mean values from three independent experiments ± SE (more than 400 cells counted). ***P<0.001. The EV1 capsid proteins were visualized after 7 h p.i. Bars, 10 µm.

Previously it has been shown that α2β1 integrin is located in the cell membrane in cholesterol-rich, detergent-resistant domains [9]. Here it was further verified that cofractionation of α2β1 integrin with caveolin-1 is not due to over-expression of integrin in SAOS-α2β1 cells but also found in other cells expressing endogenous amounts of this integrin, namely A549 cells and MDA-MB-231 cells (Figure S1A). Since modulation of the plasma membrane cholesterol level may affect the ability of integrins to form clusters [36], [37], it was investigated whether disruption of plasma membrane lipid rafts would affect the internalization of α2β1 integrin subsequent to clustering by antibodies. To test this, the cholesterol sequestering drugs filipin and nystatin were used. In order to measure internalization, a differential labeling of the plasma membrane-associated and internalized pools of α2β1 integrin was performed as described before [11]. Confocal images showed that treating cells with filipin or nystatin for 30 min before integrin clustering and internalization for 2 h resulted in plasma membrane accumulation of the receptor (Fig. 1A). In control cells, α2β1 integrin was efficiently internalized and showed normal integrin accumulation in the cytoplasmic structures, whereas in nystatin- or filipin-treated cells integrin internalization was halted (Fig. 1A). Quantification of voxels of surface and internalized α2β1 integrin signal verified that cholesterol sequestering drugs caused an arrest in internalization. The ratio of the plasma membrane-associated integrin to the internalized α2β1 integrin was significantly higher in the cells treated with filipin or nystatin (19-fold and 20-fold, respectively) when compared to control internalization for 2 h without the treatment.

Since integrin internalization was strongly inhibited with filipin and nystatin, it was further tested if also EV1 infection was affected since it uses α2β1 integrin as its receptor on cell surface. Filipin and nystatin arrested EV1 infection totally when cells were preincubated 30 min with either compound (Fig. 1B). While control cells showed a high infection rate (78.4% ±1.0% SE), filipin- or nystatin-treated cells showed no or a low degree of infection (0% and 0.5% ±0.5% SE, respectively). Confocal images of EV1-infected control cells after 7 h post infection (p.i.) showed that the cytoplasm was full of newly produced viral capsid proteins, whereas in nystatin- and filipin-treated cells, the viral capsid proteins were found only at the plasma membrane. As the SAOS cells do not normally contain α2β1 integrin but have been made stably overexpressing this integrin, infection assay was also repeated with A549 and MDA-MB-231 cells expressing endogenous levels of α2β1 integrin. First, integrin levels of these cells were verified with SDS-PAGE and blotting that showed similar amounts of integrin with SAOS-α2β1 and MDA-MB-231 cells whereas A549 cells had a lower amount of α2 integrin (Figure S1B). Despite the differences in integrin levels both of these two cell lines were very sensitive to filipin and nystatin causing a total block of infection (Figure S1C). Altogether, since both drugs caused an accumulation of integrin at the plasma membrane and arrested infection, we conclude that cholesterol-rich domains are involved in endocytosis of α2β1 integrin and thus important for EV1 infection.

### Perturbing Cholesterol Biosynthesis Halts Integrin Internalization and EV1 Infection

**Figure 2:**
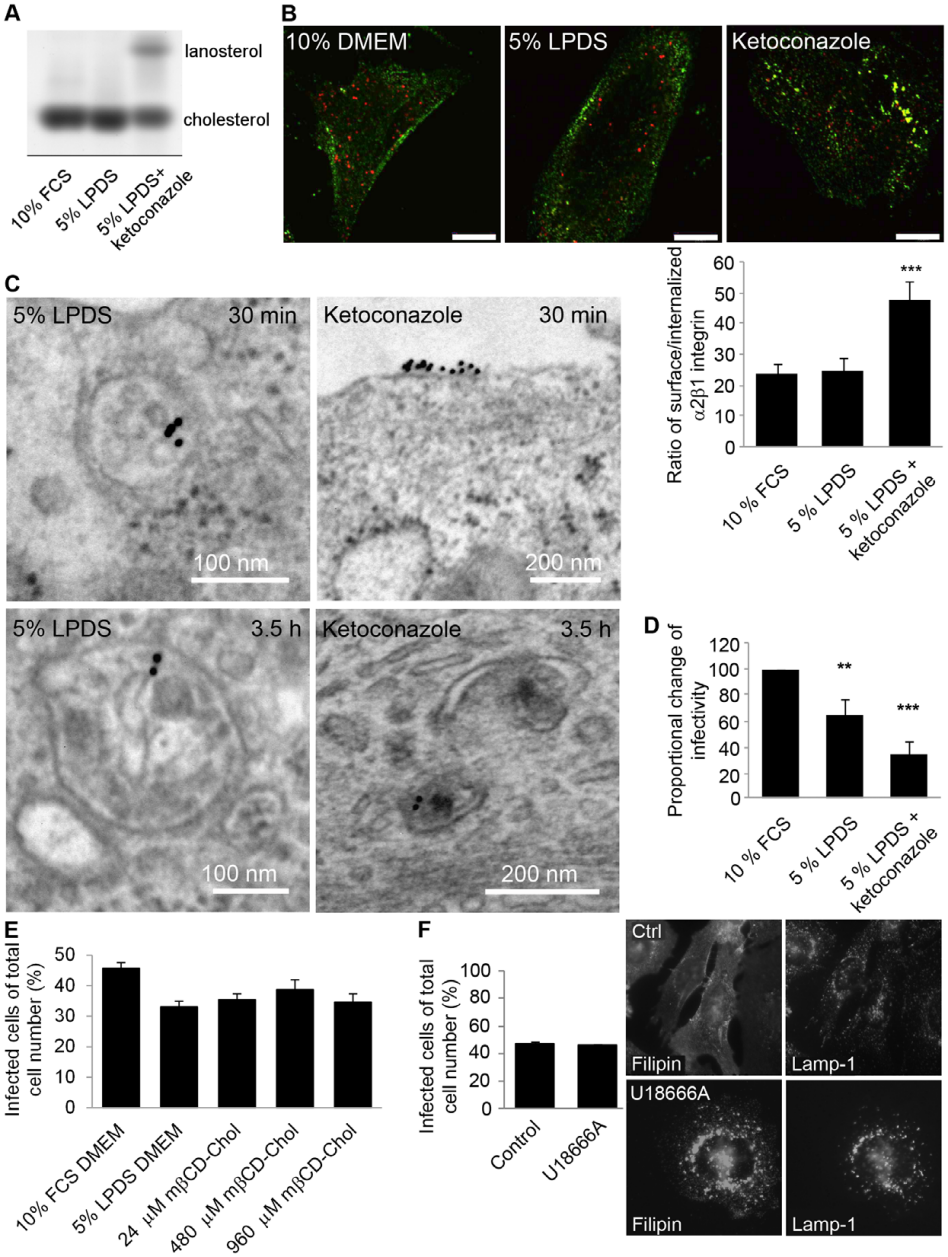
Inhibition of cholesterol synthesis disrupts integrin internalization and EV1 infection. A) Thin layer chromatography analysis of sterols in SAOS-α2β1 cells. B) Representative pictures of internalization of α2 integrin after ketoconazole, 5% LPDS or 10% FCS treatments. Internalized integrin is seen as green labeling and surface-bound integrin as red or yellow dye. The ratio of voxels between surface and internalized integrin was quantified with internalization algorithm embedded in BioImageXD software. Higher ratio means higher amount of integrin in plasma membrane. Results are averages from together 33 cells from 3 independent tests+SE. C) Electron microscopic example images of integrin structures after antibody clustering in 5% LPDS+ketoconazole cells at 0.5 and 3.5 h time points. D) Proportional change of EV1 infectivity in cells treated with 10% FCS DMEM, 5% LDPS DMEM or 5% LPDS DMEM with ketoconazole. Results are averages of four independent tests (+SE). **P<0.05, ***P<0.001. E) EV1 infectivity in cells treated with 10% FCS DMEM, 5% LDPS DMEM or 5% LPDS+mβCD-cholesterol. Results are averages of three independent tests (+SE), together over 750 cells were counted. F) The effect of U18666A (3 µg/ml) on EV1 infection percentage. EV1 infectivity was calculated together from 750 cells from three individual tests (+SE). Cholesterol labeling with filipin and lysosomal labeling with Lamp-1 was performed to confirm the efficacy of the drug. Bars 10 µm.

Next it was assessed whether inhibition of cholesterol synthesis interferes with the α2 integrin internalization pathway. To this end, we used ketoconazole, which by inhibiting the enzyme CYP51, prevents cholesterol biosynthesis at the level of the earliest sterol precursor, lanosterol. The efficacy of cholesterol depletion was monitored by a biochemical assay. Ketoconazole treatment for 3 days caused a 37% decline in cholesterol content and an equal increase of lanosterol in SAOS-α2β1 cells (Fig. 2A). In addition, cultivation of cells in lipoprotein-deficient serum (LPDS) conditions for 3 days resulted in 13% depletion of cholesterol compared to the cells grown in the presence of normal serum (Fig. 2A). When integrin was clustered under these conditions a clear inhibition in integrin internalization was observed (Fig. 2B). In normal lipoprotein conditions (10% FBS in DMEM) and also in lipoprotein-deficient conditions (starvation for 3 days in 5% LPDS in DMEM) integrins were internalized normally (Fig. 2B). In contrast, ketoconazole treatment for 3 days (in 5% LPDS, DMEM) caused the accumulation of integrin on the plasma membrane. This was verified by quantification of the ratio of integrin voxels between plasma membrane and internalized integrin. Results revealed a 2-fold enrichment of integrin label on the plasma membrane in ketoconazole-treated cells compared to 10% DMEM control (Fig. 2B). The inhibition of internalization was not as total as with filipin treatment, which caused a roughly 20-fold enrichment of integrin on the plasma membrane (Fig. 1A). This was expected as after ketoconazole treatment, 63% of cholesterol remained in cells (Fig. 2A).

In order to study if α2 integrin colocalization with detergent-resistant membranes was changed during ketoconazole treatment, we performed two different labelings. First, we labeled the raft membranes with the pore-forming toxin aerolysin conjugated to Alexa 546 as we have described before [10] (Figure S2A). This toxin binds to all GPI-anchored proteins on the plasma membrane and is thus a good marker for raft membranes. α2 integrin colocalized rather well with aerolysin in normal conditions but, interestingly, after ketoconazole treatment, both integrin and aerolysin labeling showed differences to control labeling. Integrin colocalization with aerolysin was much weaker suggesting that integrin was less abundant in the rafts, possibly contributing to the ketoconazole phenotype. Cholesterol depletion also decreased strong integrin and aerolysin labeling in the cell boundary and both labels were more scattered on the plasma membrane. Secondly, we labeled plasma membrane cholesterol by filipin staining. This labeling showed also less colocalization after ketoconazole treatment (Figure S2B). In addition, a further treatment with cold Triton X-100 caused depletion of integrin labeling after ketoconazole treatment suggesting that integrin had moved out from the detergent-resistant domain to Triton X-100 soluble domain on the plasma membrane.

α2 integrin distribution after ketoconazole treatment was further studied in detail with electron microscopy. The micrographs revealed that lipoprotein starved cells showed structures that were already partially multivesicular after 30 min internalization as we had observed before for normal biogenesis of α2-MVBs (Fig. 2C) [11] whereas in ketoconazole-treated cells integrin was found mainly on plasma membrane. After 3.5 h, lipoprotein-starved cells contained α2-MVBs with increasing amounts of intraluminal vesicles and internal membranes compared to 30 min time point as has been shown for α2 integrin internalization pathway previously [11], [15]. In contrast, no integrin containing multivesicular structures were observed in ketoconazole-treated cells, and the few found cytoplasmic structures showed tubular elements that are characteristic of the earliest forms of endosomes after integrin entry, suggesting that the biogenesis of multivesicular structures was halted (Fig. 2C) [11].

In addition to a block in α2 integrin uptake, ketoconazole treatment caused a 65% inhibition of EV1 infection compared to control cells (grown in 10% FBS DMEM) further confirming the importance of cholesterol for EV1 infection (Fig. 2D). It is also notable that cultivation of cells in lipoprotein-deficient conditions for 3 days caused a 35% reduction in infection (Fig. 2D). These results were also verified with the A549 cell line endogenously expressing α2β1 integrin (Figure S2C). Thus next it was tested if additional cholesterol could restore normal EV1 infectivity in LPDS-treated cells. LPDS-treated cells were incubated with three different doses of methyl-β-cyclodextrin (mβCD)-cholesterol complex 30 min prior to infection. Medial dose of cholesterol (480 µM) increased infection to some extent but could not totally recover infection to the normal level (Fig. 2E). Cholesterol labeling with filipin verified that lipoprotein deficient conditions for 1 day lowered the cholesterol level of cells (Figure S3A). Furthermore, 30 min treatments with 480 and 960 µM cholesterol were able to overload cells with cholesterol (Figure S3A).

3-β-[2-(diethylamino)ethoxy]androst-5-en-17-one (U18666A) is a drug that induces cholesterol accumulation to late endosomal/lysosomal membranes [38]. U18666A has been shown to perturb late endosomal function and e.g. HIV infection by inhibiting cholesterol trafficking from late endosomes to the other cellular structures [29], [39], [40]. Here, it was studied if U18666A caused any difference to EV1 infection. We have previously shown that the α2-MVBs are separate from the acidic late endosomes and they seem to stay in separate pathways even after longer internalization periods [14], [15]. As expected, U18666A treatment caused a clear accumulation of cholesterol in the Lysosomal-associated membrane protein 1 (Lamp-1) containing late endosomes/lysosomes (Fig. 2F). U18666A also had a clear effect on Lamp-1 labeling pattern causing its accumulation close to the nucleus. However, U18666A treatment did not have any effect on EV1 infection. This agrees with our earlier observation that EV1 infection is not associated with Lamp-1 containing endosomes [14].

In addition to cholesterol, lipid rafts are rich in sphingolipids. To test the importance of sphingolipids along the α2β1 integrin internalization pathway, the effect of Fumonisin B_1_, the inhibitor of sphingolipid biosynthesis, was tested. The cells were preincubated with Fumonisin B_1_ (20 µg/ml) for 48 h before infection and the compound was present also during virus internalization. The results showed that Fumonisin B_1_ caused a small but significant decline (15%) in EV1 infection (Figure S3B). This indicates that interference of sphingolipid metabolism also affects EV1 infection and taken together with earlier findings, this is likely to occur at the internalization stage.

### Uptake of Soluble Collagen is Sensitive to Cholesterol Perturbation

**Figure 3:**
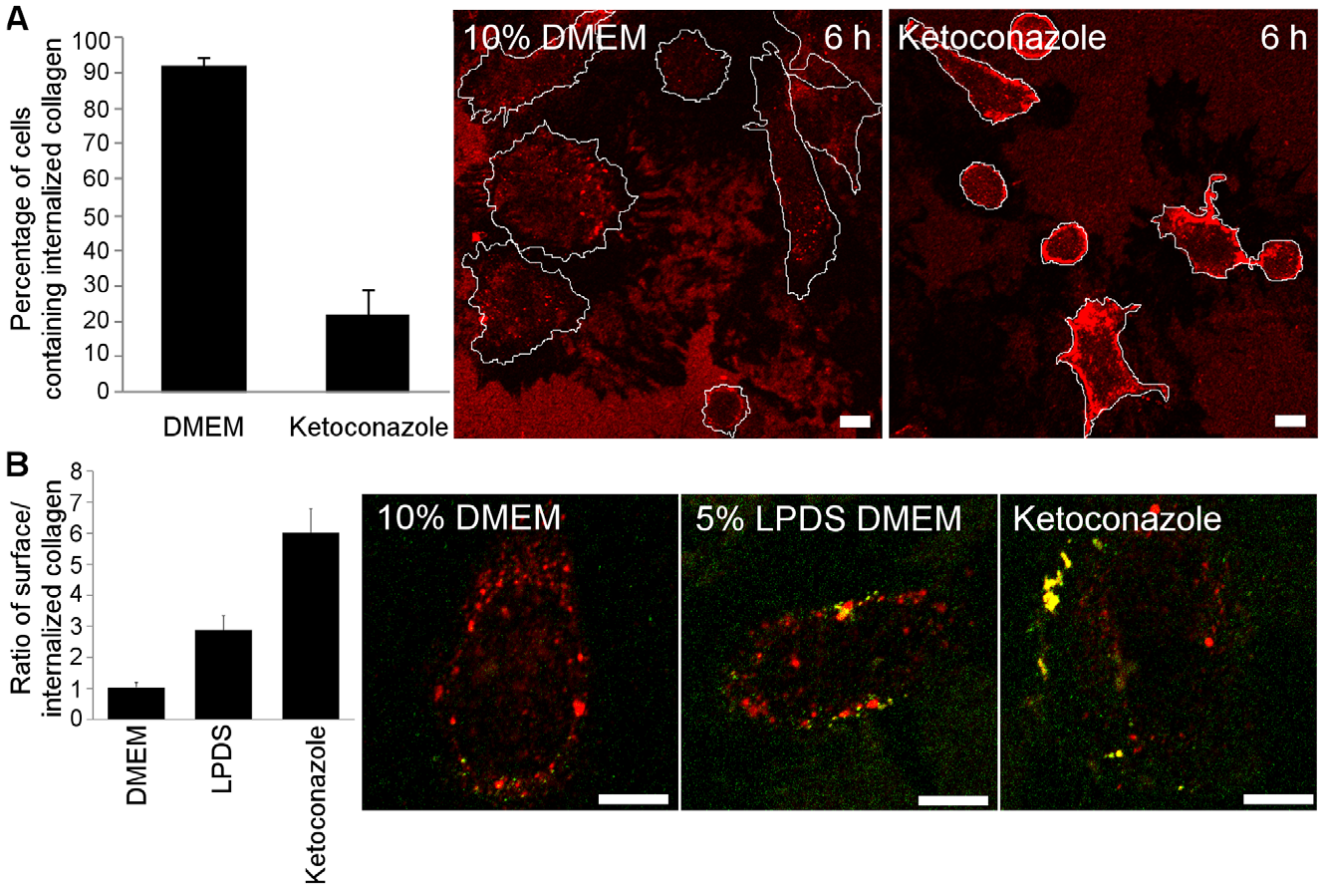
Collagen uptake is disturbed by ketoconazole treatment. A) Percentage of cells showing internalized collagen vesicles vs. cell surface-enriched collagen was counted from 77 to 80 cells cultivated in 10% DMEM or 5% LPDS DMEM with ketoconazole, respectively. Typical images used in calculations are shown. Bars 10 µm. B) Ratio of cell surface-enriched vs. internalized collagen was calculated from confocal sections from 20 cells cultivated for 6 h in 10% DMEM, 5% LPDS or 5% LPDS with ketoconazole. Surface-labeled collagen is seen as green or yellow and internalized collagen as red staining. Representative images are shown. Higher ratio means higher collagen label at plasma membrane. Results are mean values from together 20 cells of 2 independent tests (+ SE). Bars 10 µm.

Our previous results have shown that the uptake of soluble collagen triggers integrin internalization pathway, which is sensitive to calpain inhibition in a manner similar to EV1 [14]. It seems plausible that EV1 exploits the internalization pathway of soluble collagen for its own entry. We thus wanted to examine if ketoconazole treatment affected collagen and integrin uptake as well. The cells were first treated with ketoconazole for 3 days and then plated on collagen-coated coverslips. Total collagen was labeled in cells after 6 h in order to see the overall effect on collagen uptake (Fig. 3A). In ketoconazole-treated cells, collagen showed a strong accumulation on the cell surface. In contrast, the cells plated on normal medium showed collagen vesicles in the cell cytoplasm and no clear accumulation on the cell surface. Calculation of the number of cells showing the cell surface phenotype vs. cells with cytoplasmic vesicles showed an almost 5-fold difference between ketoconazole treatment and control (Fig. 3A). A more detailed labeling of collagen before and after permeabilization was then performed in order to reveal the extent of internalization. In control cells, 6 h after plating, collagen was already largely in cytoplasmic vesicles and only little cell surface signal was found (Fig. 3B). Cells starved with 5% LPDS medium showed more collagen on the plasma membrane suggesting that reduced lipoprotein content in the medium was not optimal for internalization. The ketoconazole-treated cells showed strikingly, roughly 5 times more collagen on the plasma membrane. This result is in good agreement with the measurement of cell surface vs. cytoplasmic collagen labeling.

**Figure 4:**
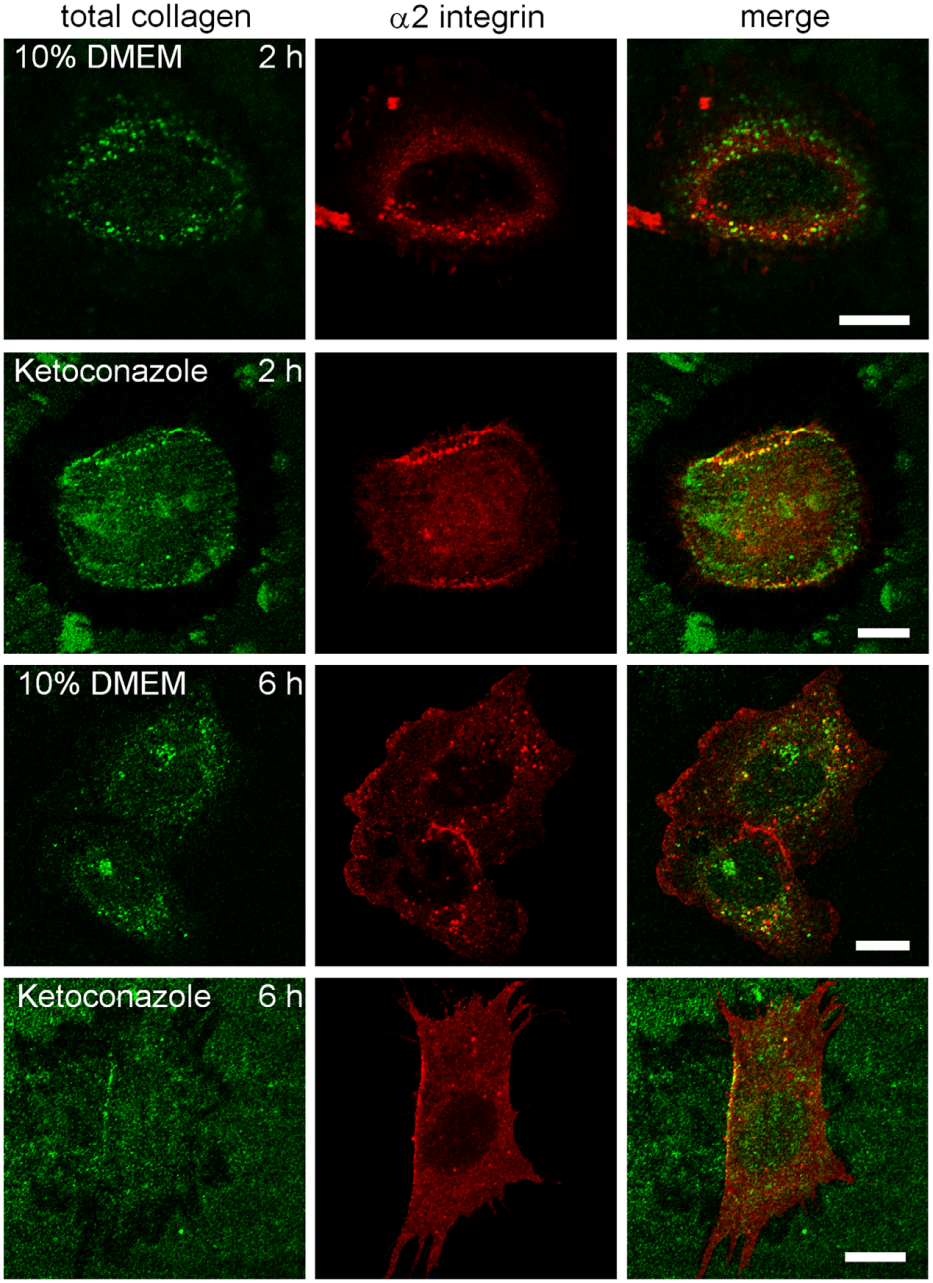
Colocalization of α2 integrin with collagen. Representative images of cells showing α2 integrin (red) and collagen (green) labeling, 2 or 6 h after plating cells on soluble collagen with or without ketoconazole. Bars 10 µm.

Collagen containing vesicles were then further characterized at 2 and 6 h after plating cells on collagen. In addition to labeling of surface and cytoplasmic vesicles containing collagen (total collagen), α2β1 integrin was also labeled. After 2 h, under normal medium conditions, part of the internalized collagen containing vesicles colocalized with α2β1 integrin indicating that α2β1 integrin cointernalized with collagen to endosomes (Fig. 4). In parallel, the α2β1 integrin had changed from a typical diffuse plasma membrane staining to a more vesicular staining pattern. After ketoconazole treatment, the collagen signal was enriched in the cellular periphery suggesting that internalization was halted. These collagen-rich areas also contained a strong α2β1 integrin signal, suggesting that the surface arrested collagen blocked α2β1 integrin on the plasma membrane. After 6 h, the control cells showed internalized collagen that partly colocalized with integrin intracellularly. Ketoconazole-treated cells showed a similar labeling after 6 h as after 2 h showing that the internalization of collagen or integrin was halted.

In conclusion, the use of the cholesterol synthesis inhibitor ketoconazole showed that a normal cholesterol environment is required for collagen uptake and intracellular accumulation together with α2β1 integrin.

### α2-MVBs are Enriched in Detergent-resistant Membranes

**Figure 5:**
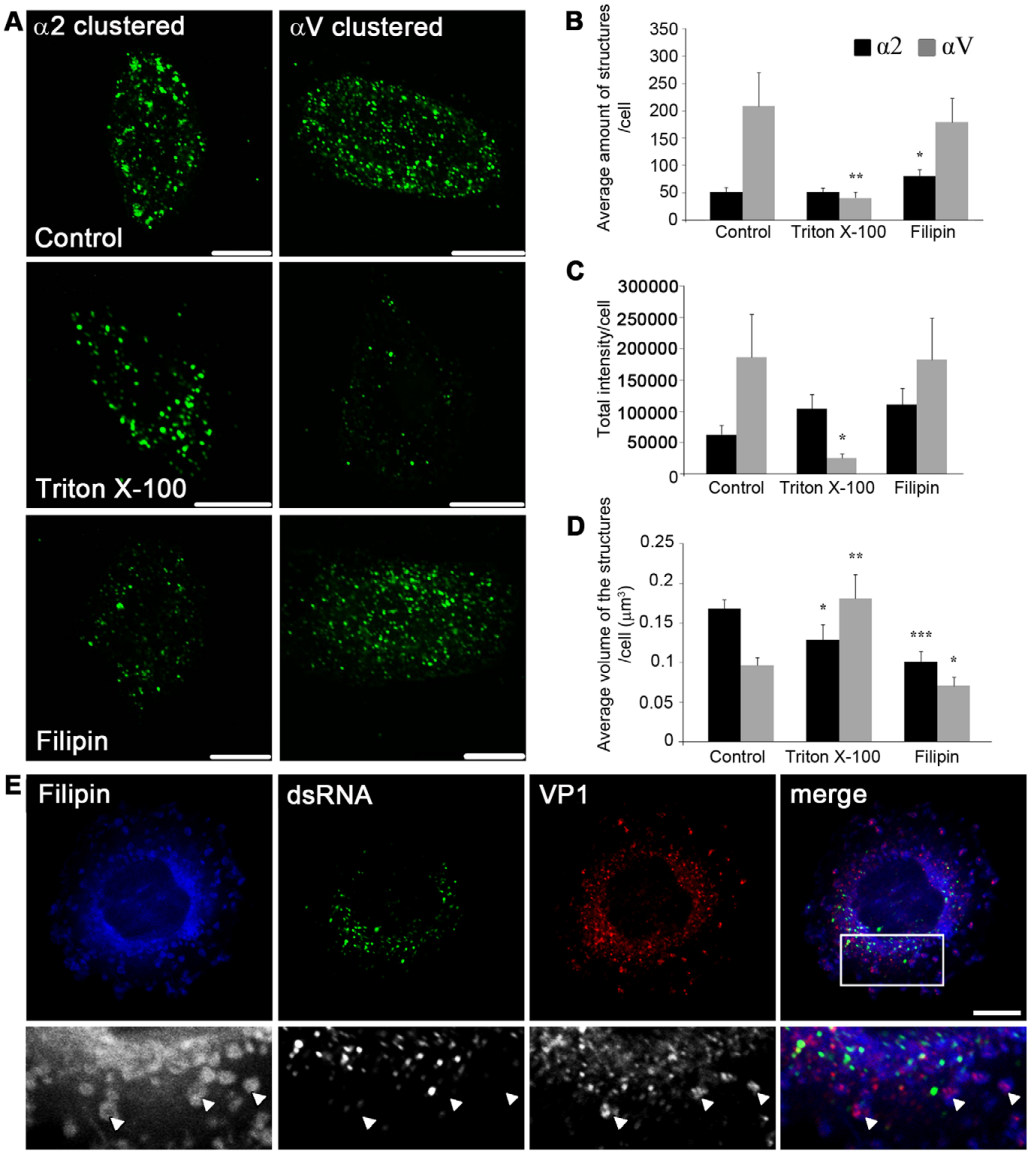
α2 integrin structures contain lipid microdomains. A) Confocal images of control cells and cells treated with Triton X-100 or filipin 2 h after clustering and internalization. Bars, 10 µm.B) The average amount of structures positive for α2 or αV integrin per cell. C) The sum of average fluorescence intensities of structures positive for α2 or αV integrin. D) The average volume of α2 or αV integrin-positive structures per cell. B–D) The results were quantified with the segmentation tool in the BioImageXD software. ***P<0.005, **P<0.001, *P<0.01. Results are expressed as mean values measured from z-stacks of 15 cells ± SE. E) Representative confocal image of EV1 infected cell at 4 h p.i. labeled with filipin (blue), anti-dsRNA (green) and anti-VP1 (red) antibodies. Illustrative examples of filipin and VP1 colocalizing structures are marked with arrowheads. Bar 10 µm.

Next it was investigated whether the membranes formed following α2β1 integrin clustering and internalization (α2-MVBs) are resistant to detergent extraction. Detergent-resistant membranes composed of high amounts of cholesterol and sphingomyelin have been defined by their insolubility in ice-cold Triton X-100 [41]. In these experiments, clustered αV integrin was used as a control, since it uses a different endocytosis route than clustered α2 integrin. Internalized αV integrin colocalizes with EEA1 and transferrin receptor after internalization in contrast to α2β1 integrin which does not colocalize with these markers [10]. Quantification of αV integrin internalization after 2 h of clustering showed that the majority of clustered αV integrin resided in intracellular structures [10] as is the case also for clustered α2β1 integrin (Fig. 1). However, the internalized αV integrin showed more numerous and smaller vesicular structures throughout the cytoplasm compared to internalized α2 integrin (Fig. 5A, D). Treatment of cells with cold Triton X-100 treatment for 30 min on ice after 2 h internalization period did not have any apparent effect on the number or intensity of α2 integrin-positive structures in cells (Fig. 5A–C). Similar results were observed in A549 and MDA-MB-231 cells (Figure S4A). Observations in SAOS-α2β1 cells were verified by quantification of the fluorescence signal using the segmentation tool in the BioImageXD software (Fig. 5B, C). In contrast, the amount of αV integrin-positive structures was greatly lowered (80.2% ±5.2% SE) in cells treated with Triton X-100 (Fig. 5B). Also the average fluorescence intensity was reduced by 86.0% ±3.7% SE in αV integrin-positive structures (Fig. 5C). The size of both α2 and αV integrin-positive structures was, however, affected by the Triton X-100 treatment: α2 integrin-positive structures were slightly smaller (23.6% ±11.6% SE reduction in average size) whereas the few αV integrin-positive structures that were left in the cytoplasm were larger (87.8% ±31.3% SE larger) compared to the structures in control cells (Fig. 5D).

Since α2-MVBs were not affected by Triton X-100 the effects of cholesterol sequestering drugs were next tested on those structures. Treatment with filipin resulted in an increase (55.1% ±22.6% SE) in the amount of α2 integrin-positive vesicles compared to control cells (Fig. 5B). The mean fluorescence intensity of α2 integrin-positive structures was not significantly affected by filipin (Fig. 5C), but the size of α2 vesicles was reduced by 40.0% ±7.7% SE compared to the control cells (Fig. 5D), indicating that the α2 integrin structures were significantly affected by filipin. The calculated average size of αV integrin structures was also reduced by filipin but to a lesser extent (26.6% ±11.3% SE) (Fig. 5D).

To more directly address if the EV1-α2β1 integrin domains in endosomes are enriched in cholesterol, the cellular cholesterol was labeled with filipin (Fig. 5E, Figure S4B). To find out if EV1 replicative structures are enriched in cholesterol, the cells were labelled after 4 h of infection, when the virus capsid proteins are still in endosomes but the viral replication has already started. We have earlier shown that the virus capsids stay with clustered integrin in α2-MVBs whereas the RNA replication mainly occurs outside the α2-MVBs in the cytoplasm [7]. Here, it was found that the virus capsid protein VP1-positive α2-MVBs vesicles were frequently positive for filipin (Fig. 5E). In contrast, the replication structures that were labeled with antibodies against the dsRNA, showed negligible labeling with filipin (Fig. 5E).

The above results thus suggest that the α2-MVBs are enriched with cholesterol containing detergent-resistant membranes and are affected by cholesterol sequestering drugs.

### Cholesterol Aggregating Drugs Inhibit EV1 Infection and Uncoating via Direct Effects on α2-MVBs

**Figure 6:**
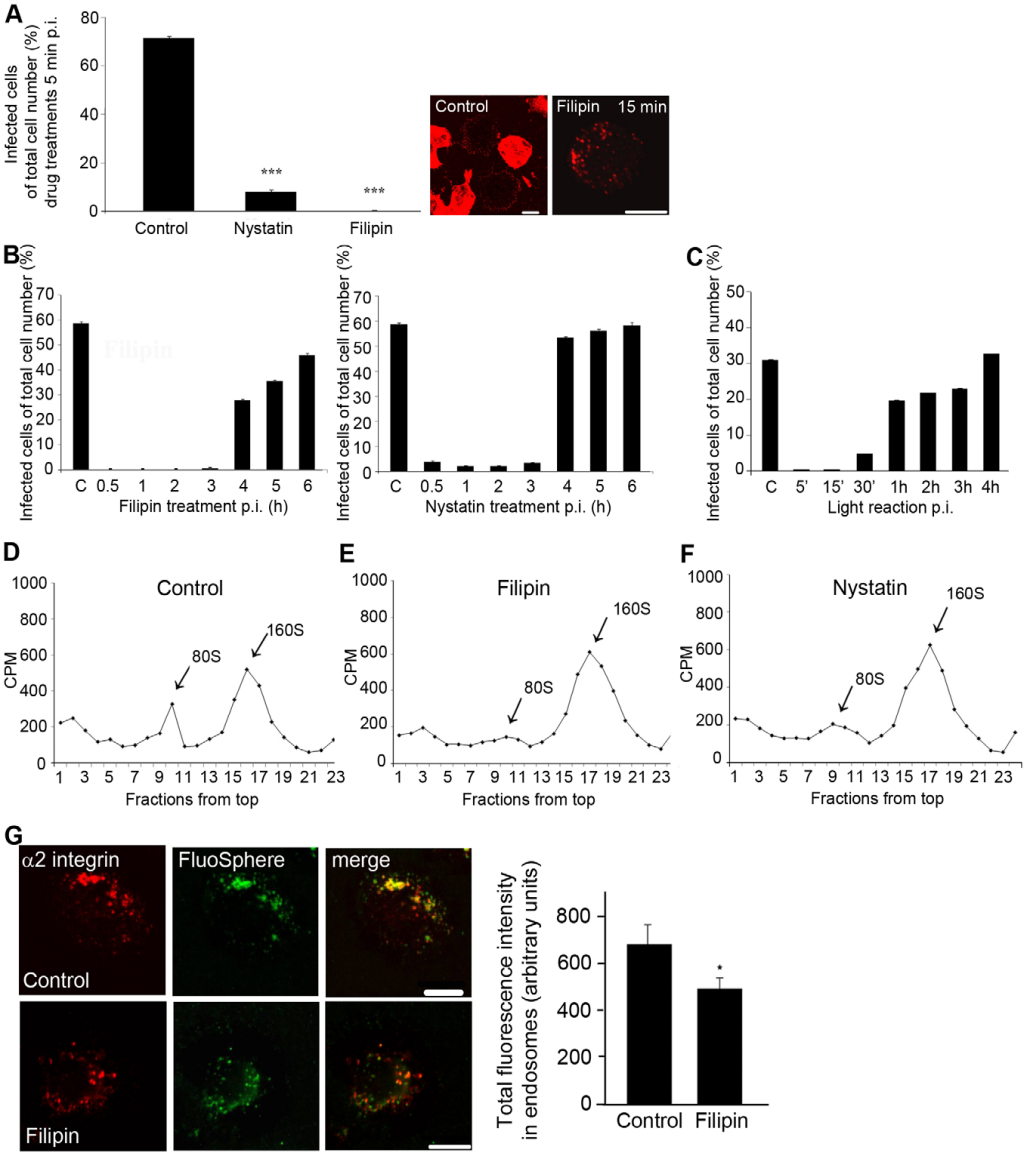
The effects of cholesterol aggregating drugs on EV1 uncoating. A) EV1 infectivity of control cells and cells treated with filipin and nystatin after 5 min p.i. (P<0.001, binomial *t*-test). Confocal images show labeled EV1 capsid proteins at 7 h p.i. Bars, 10 µm. B) EV1 infection percentage of cells treated with nystatin or filipin that were added at different time points p.i. The results are mean values of 3 independent experiments ± SE (more than 700 cells counted). C) Infection percentage of neutral-red labeled EV1 (NR-EV1) with light treatments in different times p.i. The control cells were not exposed to light reaction. Results are averages of 2 independent tests (+ SE) and over 800 cells were counted at the minimum. D) Sucrose gradient sedimentation assay of uncoating with [^35^S]methionine-labeled EV1 at 4 h p.i. RNA containing virus sediments at 160S, whereas the 80S represents empty capsids from which the viral RNA genome is released. E-F) As in D except filipin or nystatin was added to the cells 15 min p.i. G) Cointernalized Fluospheres and clustered α2 integrin colocalize in endosomes. Total intensity of FluoSpheres in endosomes was analysed from confocal sections by using the intensity algorithm in BioImageXD. Together 20 cells from 2 separate tests were analysed (+ SE). *P<0.01. Bars, 10 µm.

Since the α2-MVBs were found to be cholesterol-rich, we studied the functional effects of the cholesterol sequestering drugs on α2-MVBs by measuring their effects on EV1 uncoating and on the appearance of newly formed EV1 capsid proteins. Virus, first bound on ice, was allowed to internalize at +37°C for various times before drug additions. Nystatin or filipin were added between 5 min and 6 h p.i., and the infection was allowed to proceed for up to 7 h. At 5 min p.i., the drugs inhibited the infection in a similar manner as when the cells were preincubated with filipin or nystatin (Fig. 6A), probably because many of the macropinocytic structures were still connected to the plasma membrane. Our previous EM studies have shown that EV1 becomes largely associated with MVBs already after 15 to 30 min p.i. [11], [15]. When filipin was added between 30 min and 3 h p.i., it still inhibited EV1 infection almost completely (1 h p.i. 0%, 2 h p.i. 0%, 3 h p.i. 0.38% ±0.11% SE) (Fig. 6B). A lower dose of filipin (0.75 µg/ml) allowed some infection when added from 2 h onwards but still showed a total block of infection between 5 min to 1 h, suggesting that the earliest time points are most sensitive to filipin action (data not shown). Nystatin also had a strong inhibitory effect on the virus infection, even though it allowed some cells to be infected (Fig. 6B). When the drugs were added at 4 h p.i., a significantly higher rate of infection was observed after filipin (27.9% ±0.44% SE) and nystatin (53.5% ±0.46% SE) treatments, suggesting that the drugs did not directly inhibit the replication of the virus. Our previous results using real-time PCR have shown that EV1 replication starts around 3 h p.i. [42]. These results thus indicate that EV1 needs intact cholesterol containing membranes to internalize and efficiently infect cells. In addition, the data suggest that the virus is dependent on the intact cholesterol containing domains in α2-MVBs until the virus genome is released from them for replication. The results also showed that filipin and nystatin do not interfere with the replication of the virus and formation of new virus particles.

Our previous results suggested that the EV1 RNA genome is released directly from α2-MVBs into the cytoplasm for replication [7]. The viral uncoating is likely to occur well before the RNA genome is released from the α2-MVBs, which occurs between 2.5 h and 3 h p.i., around the time when the replication starts in the cytoplasm [42]. In order to first evaluate the time table of uncoating, we used the neutral red labeling technique [43]. Neutral red-labeled virus is light-sensitive and, upon exposure to light, the dye causes cross-linking of the labeled genome, thus inhibiting virus uncoating. If the virus has not undergone uncoating before the light reaction, there will be no newly synthesized virus in the assay. On the other hand, the uncoated RNA outside the virus particle will promote infection despite of the later light reactions. When control cells, infected with neutral-red labeled EV1, were treated with a strong light for 10 min at 5 or 15 min p.i., there were no infected cells detected after 7 h, suggesting that no virus had uncoated at those time points (Fig. 6C). However, after 30 min p.i., 4.9% ±0.07% SE of the cells were infected, suggesting that uncoating had started. Later, at 1 to 4 h p.i., around 20–30% of the cells was infected, reaching the infection level of the control cells (no light treatment; 31.1% ±0.15% SE) (Fig. 6C).

To test whether cholesterol aggregating drugs had an effect on EV1 uncoating, the cells were treated with [^35^S]methionine-labeled EV1 and the viral uncoating was analysed by sucrose gradient sedimentation. Radiolabeled EV1 particles were bound to cell surface for 1 h on ice, followed by incubation at +37°C for 15 min before the drugs were added, to allow internalization of the virus. The infection was then allowed to proceed for 4 h after which the cells were collected and the sedimentation of cell-associated virus was analysed in sucrose gradients. EV1 containing RNA sediments at 160S, whereas the release of the viral RNA genome leads to the formation of empty capsids, which sediment at 80S [9]. Our results showed that in the control infection 19% of the virus particles were uncoated after 4 h (Fig. 6D).The viruses from the cells treated with filipin still contained the RNA (160S intact form) and only a small fraction of viral particles (80S form) were seen in the light fractions in the gradient (Fig. 6E). Quantification of the ratio between the 160S and 80S forms showed that only 4.9% of EV1 was uncoated after filipin treatment. Results with nystatin were similar, with a small peak of uncoated 80S viruses (9.7%) being detected (Fig. 6F). This is in line with our previous results from EV1 infection experiments (Fig.s 6B) suggesting that nystatin is not quite as effective as filipin in inhibiting EV1 entry and infection.

Polyene antibiotics, like nystatin and amphotericin B, are generally used in patch clamp technique to induce permeability of ions of cellular membranes [44], [45]. Therefore it was further studied whether the block in uncoating was at least partially due to increased permeability in the structures. In order to verify if the drugs indeed broke some of the structures, we co-internalized the fluid-phase marker FluoSpheres with the clustered integrin (Fig. 6G). Confocal microscopy of the control samples revealed that the FluoSpheres colocalized well with internalized clustered integrin (Fig. 6G). When filipin was added to cells at 1 h time point, 2 h incubation led to 28% reduction of the FluoSphere intensity level of the endosomes (Fig. 6D). This indicates that FluoSpheres were partially able to leak out from the cholesterol-aggregated structures, suggesting that some small openings formed during the filipin treatment.

Altogether these findings show that cholesterol aggregating drugs have direct effects on the cytoplasmic α2-MVBs leading to their permeabilization, decreased virus uncoating and reduced infection.

## Discussion

**Figure 7:**
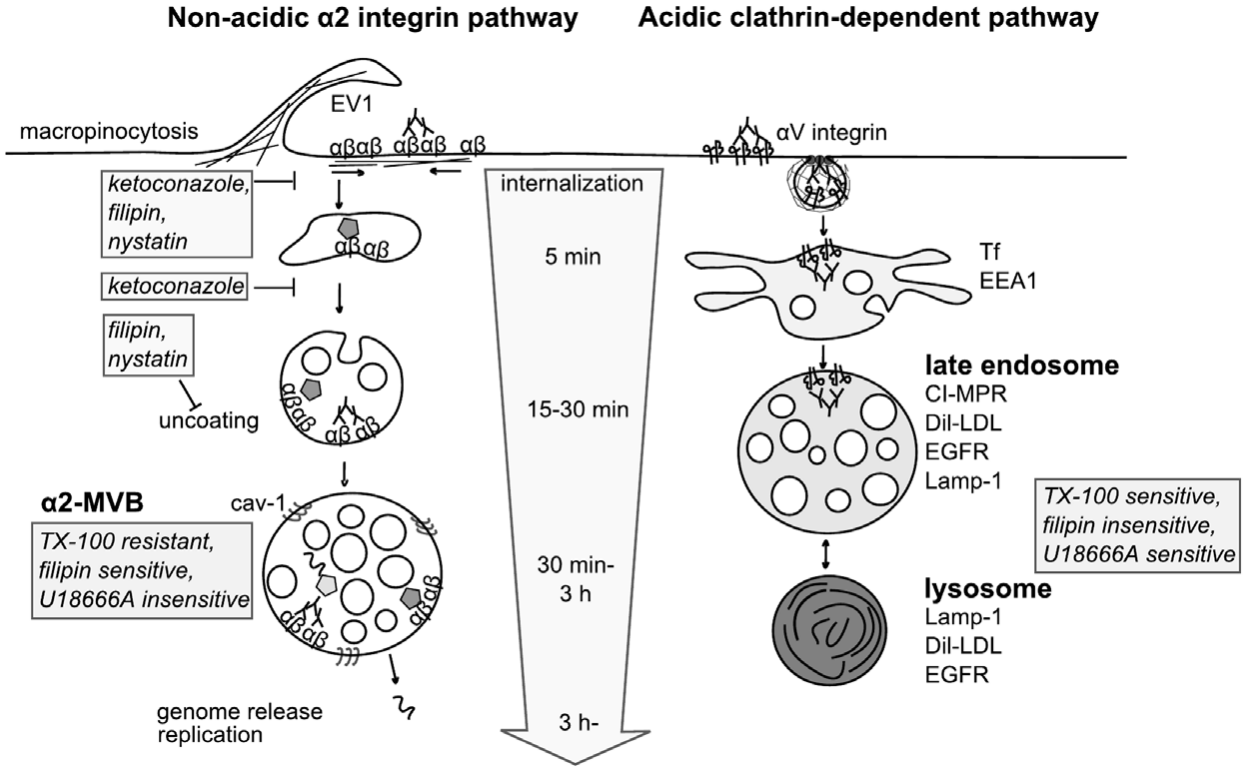
Summary of the non-acidic α2 integrin clustering-triggered and the acidic clathrin-dependent pathways. EV1 or antibody clustering triggers integrin internalization via macropinocytosis and leads to formation of multivesicular structures that are distinct from clathrin-dependent internalization and conventional acidic endosomes and lysosomes [10], [14], [15]. Pertubation of cholesterol with filipin, nystatin or ketoconazole inhibited internalization of α2 integrin and its ligands. Furthermore, the maturation of α2-MVBs was also prevented after cholesterol depletion. α2 integrin structures were found to be rich in cholesterol based on several criteria: 1) cholesterol sequestering drugs inhibited the viral uncoating inside the endosomes, 2) α2-MVBs were resistant for Triton X-100 treatment and sensitive for filipin in contrast to αV integrin structures, and 3) EV1 containing structures were filipin positive. Moreover, EV1 infection was insensitive for U18666A whereas the compound interferes with trafficking from classical late endosomes and lysosomes. Abbreviations used: α2-MVB, α2 integrin containing multivesicular bodies; cav-1, caveolin-1; CI-MPR, cation-independent mannose-phosphate receptor; Dil-LDL, 1,1′-dioctadecyl-3,3,3′,3′-tetramethylindocarbocyanina-labeled LDL; EEA1, early endosomal antigen 1; EGFR, epidermal growth factor receptor; EV1, echovirus 1; Lamp-1, Lysosomal-associated membrane protein-1; Tf, transferrin; TX-100, Triton X-100; U18666A, 3-β-[2-(diethylamino)ethoxy]androst-5-en-17-one.

The novel α2β1 integrin clustering triggered internalization pathway starts from the lipid microdomains on the plasma membrane and shows caveolin-1 accumulation in the forming intracellular multivesicular structures [10], [11]. In this study, we investigated whether the lipid microdomains and especially cholesterol play a role also after ligand uptake, during virus uncoating in endosomes and in the biogenesis of the non-acidic multivesicular bodies. A schematic presentation of the main findings is presented in Fig. 7.

We show here that the uptake of clustered α2β1 integrin is strictly dependent on intact cholesterol domains on the plasma membrane. EV1, integrin and soluble collagen were all largely blocked on the plasma membrane if we caused aggregation of cholesterol or if we lowered the cellular levels of cholesterol by preventing its biogenesis. It has been suggested that cholesterol has a crucial role in the assembly of integrin-associated protein signaling complexes [36]. We have shown recently that integrin clustering induced internalization relies on activation of PKCα, PLC, PI3K and Rac1, and that inhibition of any of those regulators causes a halt in internalization [10], [11]. It remains thus possible that inhibition of efficient assembly of these signaling proteins or inhibition of activation of any of these factors could be contributing to the block in uptake. Cholesterol-rich membrane domains have been shown to mediate entry of enveloped flaviviruses such as dengue virus and Japanese encephalitis virus [46], [47]. In addition, filipin was shown to block HIV gag protein entry and translocation to MVBs in a clathrin-independent pathway [48]. We found here that cholesterol domains were also needed for the biogenesis of cytoplasmic EV1-induced multivesicular bodies. Ketoconazole treatment, which caused accumulation of lanosterol and lowered the amount of cholesterol in cells, caused integrin depletion out from detergent-resistant domains and had a drastic effect on internalization. The endosomal structures that were observed in the cytoplasm were early endosomal type tubulovesicular structures, suggesting that sufficient content of cholesterol in the membranes is needed for the formation of multivesicularity. In line with these results, the deletion of yeast *OSH* genes encoding oxysterol-binding proteins alters intracellular sterol distribution and causes vacuolar fragmentation [49]. On the other hand, overexpression of one sterol lipid carrier ORP1L was shown to cause enlarged multivesicular bodies with higher amount of internal membranes [50] altogether suggesting that sterol abundance and distribution is important for the biogenesis and function of endosomes.

We showed further that cholesterol-rich domains are co-internalized and exist in the formed α2-MVBs. This was proven by cholesterol labeling with filipin and by the classical cold Triton X-100 treatment, which had no solubilizing effect on the α2-MVBs whereas it had a more pronounced effect in the control endosomal structures labeled and induced by clustered αV integrin. This was expected as we had recently shown that cholesterol binding caveolin-1 rich domains are also accumulating in α2-MVBs [11]. We also knew from our previous studies that clustered αV integrin is internalized via clathrin coated pits and colocalizing with EEA1 and transferrin unlike EV1 [10]. Although it has been shown that different endosomes contain lipid raft components [25], [26], our results suggest that α2-MVBs, originated from the cholesterol-rich plasma membrane domains contain a higher amount of detergent-insoluble lipids than the antibody clustered αV integrin structures studied here.

Experiments with filipin and nystatin clearly showed that EV1 inside α2-MVBs was sensitive to these drugs. Administration of these drugs halted virus infection at the α2-MVBs. While drug addition during the active replication period was not much affected, administration during the early time points totally prevented infection. The later replication phase has been shown to be sensitive for cholesterol perturbation for hepatitis C virus, West Nile virus and Japanese encephalitis virus [18], [19], [46]. In the case of West Nile virus, virus causes redistribution of cholesterol from the plasma membrane and recruits cholesterol to the dsRNA-positive replication sites [19]. Hepatitis C virus RNA was shown to accumulate in detergent-insoluble vesicles and to colocalize with caveolin-2 [18]. Also flaviviral non-structural proteins were shown to associate with detergent-resistant membranes [46]. In contrast, labeling of dsRNA during EV1 replication showed that indeed, dsRNA-positive replication sites did not accumulate cholesterol, further suggesting that the replication step of EV1 is not particularly dependent on membranes enriched in cholesterol. These data suggest that there are clear differences among viruses on their requirement for lipids during the viral infectious cycle. Most probably this reflects the differences in their internalization and replication strategies. This needs to be taken into account while developing new anti-viral schemes involving lipid modifying drugs.

Evaluation of the EV1 structure during endosomal trafficking indicated that filipin and nystatin treatments prevented the actual uncoating inside α2-MVBs. EV1 stayed as an intact 160 S particle while normally the conversion to the 80S starts already after 30 min. Our previous observations have suggested that, unlike many other viruses, binding of EV1 to the receptor integrin does not start the uncoating process on the plasma membrane [9]. Instead, this binding stabilizes the virus and uncoating occurs later, in intracellular endosomes, before the genome is released to the cytoplasm [7]. More careful evaluation of the uncoating timing here using the NR-EV1 showed that uncoating of EV1 starts after 30 min p.i. Several studies suggest that changes in ion concentrations may promote viral uncoating in endosomes [51], [52]. While it remains to be shown whether specific ion gradients are needed for EV1 uncoating, our results suggest that membrane permeabilization due to filipin and nystatin treatments compromised the ambient conditions needed for uncoating. Filipin has been shown to mediate permeabilization of ions [44], [53]. FluoSpheres internalization test showed that FluoSpheres content had partially leaked from the structures. This may be due to perforations large enough to leak some of the Fluosphere particles, but also partially due to possible fragmentation of the structures. Fragmentation of cellular membranes has been shown to occur after cholesterol perturbation using filipin treatment or affecting the expression of sterol binding oxysterols [49], [54]. Cholesterol overloading with moderate amount of mβCD-cholesterol increased infection to some extent but did not completely restore viral infection whereas higher amount of cholesterol had no positive effect on infection. Our results altogether suggested that cholesterol modifying drugs have direct effects on α2-MVBs with consequences on viral uncoating and infection, indicating dependence on cholesterol-rich membranes.

The entry route of soluble collagen with clustered integrin also showed great sensitivity to cholesterol disturbing conditions. This was expected as we recently discovered the similarities with the EV1 induced internalization pathway [14]. The exact fate of internalized collagen along this pathway is not yet known. How the cholesterol-rich membranes are contributing to collagen trafficking remains to be elucidated.

According to our previous results, the α2 integrin internalization pathway differs from the clathrin-dependent pathway by several criteria. α2 integrin internalization pathway 1) is not acidic, 2) does not contain markers of the conventional early and late endosomes or lysosomes, 3) is separate from EGF receptor degradation and 4) promotes integrin degradation via activation of neutral calpains (Fig. 7) [14], [15]. In this study we show the importance of cholesterol-rich membranes for the uptake of cargo such as virus and collagen along this novel integrin clustering pathway. The cholesterol containing endosomes along the α2 integrin pathway are sensitive to filipin and cholesterol depletion. Furthermore, we show that perturbation of cholesterol disturbs endosomal functions such as virus uncoating in endosomes and the biogenesis of integrin induced multivesicular bodies. However, replication of EV1 is not dependent on cholesterol and infection through these α2-MVBs is not sensitive to U18666A in contrast to the endosomal trafficking via the acidic late endosomes. The results altogether stress the importance of cholesterol on the structure and functions of the novel non-acidic multivesicular bodies.

## Supporting Information

Figure S1 **A)** **α2 integrin cofractionates with detergent-resistant membranes.** Cells from four confluent 10 cm ø dishes were subjected to sucrose flotation gradient centrifugation as described before (Upla et al. Mol. Biol. Cell 15∶625–636, 2004). After unloading the gradient, protein concentrations were determined and equal amounts of proteins from each fraction were by ultracentrifugation. Proteins were suspended at Laemmli buffer and subjected to SDS-PAGE and blotting. Blots were labeled with rabbit α2 integrin antibody (Millipore) and rabbit caveolin-1 antibody (Santa Cruz). DRM, detergent-resistant membranes. **B) Evaluation of α2 integrin levels in different cell types.** SAOS-α2β1, MDA-MB-231 and A549 cells were scraped at Laemmli buffer and subjected to SDS-PAGE and blotting. Blots were labeled with rabbit α2 integrin antibody (Millipore) and mouse tubulin antibody (Cedarlane Laboratories). **C) Filipin and nystatin block EV1 infection totally in different cell lines.** Representative images of EV1 infected SAOS α2β1, MDA-MB-231 and A549 cells pretreated with 0.75 µg/ml filipin or 50 µg/ml nystatin for 30 min. Drugs were also present during 6 h incubation after virus binding. Cell nuclei are labeled with DAPI (blue, Invitrogen) and newly produced viral capsid proteins with VP1 antibody (green, Marjomaki et al. J. Virol. 76∶1856–1865, 2002). More than 800 cells were monitored in each case.
(TIF)

Figure S2 **A) Ketoconazole treatment affects the localization of unclustered α2 integrin and aerolysin toxin.** SAOS-α2β1 cells pretreated for 3 days ±10 mM ketoconazole were labeled with mouse α2 integrin antibody (A211E10) on ice and after 4% PFA fixation with goat anti-mouse Alexa 488 (green) and Alexa 546-conjugated aerolysin toxin (ASSP, red, Fivaz et al. Embo J. 21∶3989–4000, 2002). Bars 20 µm. **B)** **Ketoconazole treatment affects the localization of clustered α2 integrin and cholesterol on the plasma membrane.** SAOS-α2β1 cells pretreated for 3 days ±10 mM ketoconazole were clustered with mouse α2 integrin antibody (A211E10) and goat anti-mouse Alexa 488 on ice. After integrin clustering cells were treated ±0.2% Triton X-100 for 30 min on ice followed by 4% PFA fixation. 0.5 mg/ml filipin was used to label cholesterol at +37°C for 30 min after fixation. **C) Ketoconazole treatment inhibits EV1 infection.** The effect of 5% LPDS and ketoconazole treatment was tested in human lung carcinoma cell line, A549 and SAOS α2β1 cells. Cells were pretreated with 10% FCS DMEM, 5% LPDS DMEM and 10 mM ketoconazole for 3 days before testing EV1 infectivity. The results are mean values of three independent samples (+ SE).
(TIF)

Figure S3 **A) mβCD-cholesterol treatment overloads cells with cholesterol.** Representative images of cholesterol loading. SAOS-α2β1 cells were grown for 1 day in 5% LPDS before mβCD-chol treatment for 30 min. 4% PFA fixed cells were labeled with 0.5 mg/ml filipin in PBS for 30 min at +37°C. Images were taken with identical wide-field microscope settings. Bar 10 mm. **B) Inhibition of sphingomyelin synthesis suppresses EV1 infection.** Cells were pretreated with 20 mg/ml Fumonisin B1 in 10% FCS DMEM for 2 days. Drug was also present in infection medium. The results are averages of two independent tests (+SE), more than 1000 cells were counted.
(TIF)

Figure S4 **A) α2-MVBs are resistant for cold Triton X-100 treatment in different cell lines.** SAOS-α2β1, MDA-MB-231 and A549 cells were clustered with α2 integrin (A211E10) and goat anti-mouse Alexa 488 antibodies on ice. After internalization for 2 hours, cells were treated ±0.2% Triton X-100 for 30 min at +4°C before fixation with 4% PFA. Bars 20 µm. **B) Filipin colocalizes with α2 integrin structures.** SAOS-α2β1 cells cells were treated with EV1 for 45 min prior α2 integrin was further clustered with sequential antibody treatments on ice (A211E10 and goat anti-mouse Alexa 488, respectively). Virus and integrin were allowed to internalize for 2 h after which cells were fixed with 4% PFA. Cellular cholesterol was labeled after fixation with 0.5 mg/ml filipin in PBS for 30 min at +37°C. Bars 10 µm.
(TIF)
