# Supplementary figures and images for: Cholesterol Dependence of Collagen and Echovirus 1 Trafficking along the Novel α2β1 Integrin Internalization Pathway
Source: PLoS One. 2013 Feb 5;8(2):e55465. doi: 10.1371/journal.pone.0055465 (PMC3564754; doi:10.1371/journal.pone.0055465)

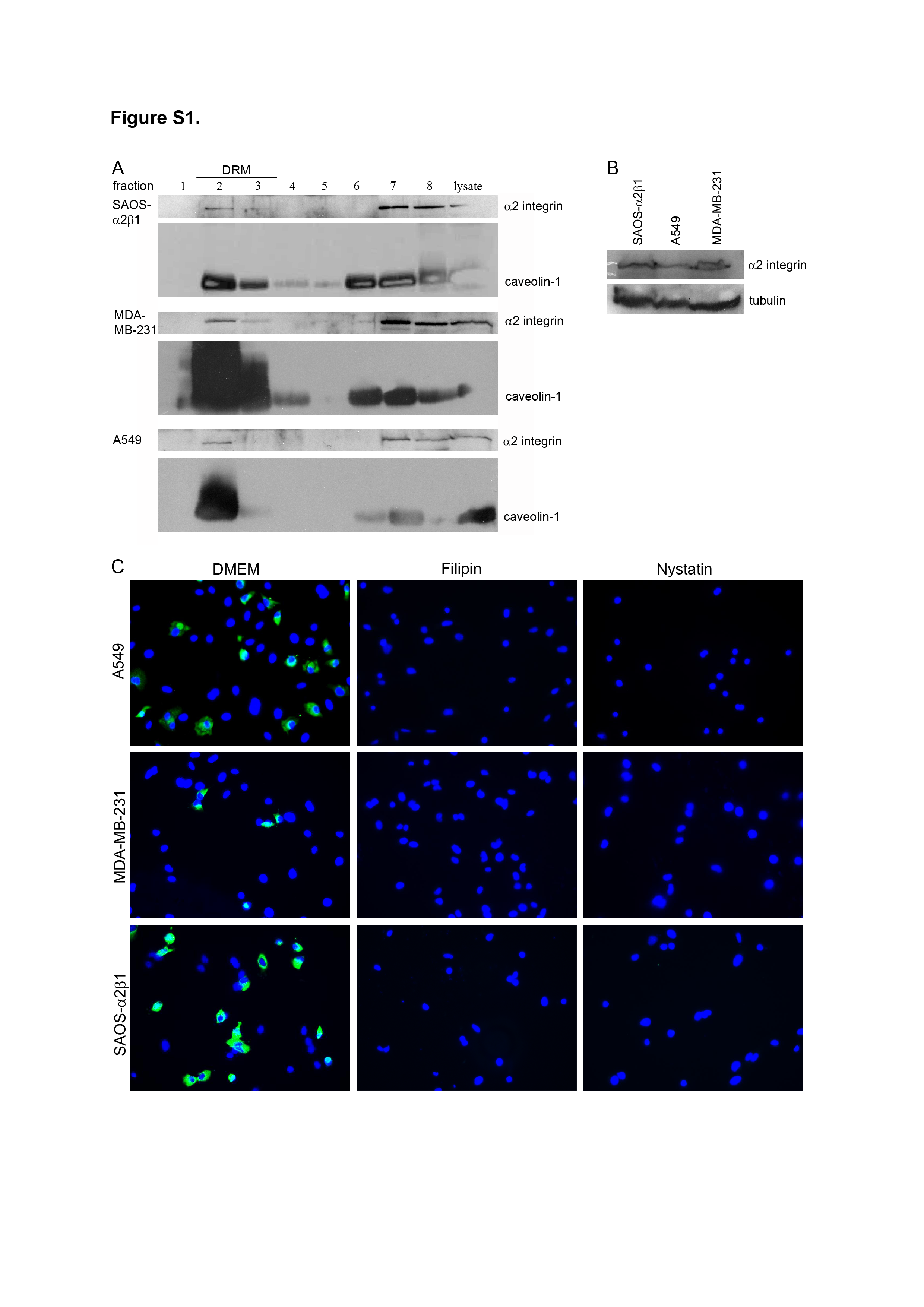

Supplement: Figure S1 — A) α2 integrin cofractionates with detergent-resistant membranes. Cells from four confluent 10 cm ø dishes were subjected to sucrose flotation gradient centrifugation as described before (Upla et al. Mol. Biol. Cell 15∶625–636, 2004). After unloading the gradient, protein concentrations were determined and equal amounts of proteins from each fraction were by ultracentrifugation. Proteins were suspended at Laemmli buffer and subjected to SDS-PAGE and blotting. Blots were labeled with rabbit α2 integrin antibody (Millipore) and rabbit caveolin-1 antibody (Santa Cruz). DRM, detergent-resistant membranes. B) Evaluation of α2 integrin levels in different cell types. SAOS-α2β1, MDA-MB-231 and A549 cells were scraped at Laemmli buffer and subjected to SDS-PAGE and blotting. Blots were labeled with rabbit α2 integrin antibody (Millipore) and mouse tubulin antibody (Cedarlane Laboratories). C) Filipin and nystatin block EV1 infection totally in different cell lines. Representative images of EV1 infected SAOS α2β1, MDA-MB-231 and A549 cells pretreated with 0.75 µg/ml filipin or 50 µg/ml nystatin for 30 min. Drugs were also present during 6 h incubation after virus binding. Cell nuclei are labeled with DAPI (blue, Invitrogen) and newly produced viral capsid proteins with VP1 antibody (green, Marjomaki et al. J. Virol. 76∶1856–1865, 2002). More than 800 cells were monitored in each case. (TIF) [file pone.0055465.s001.tif]

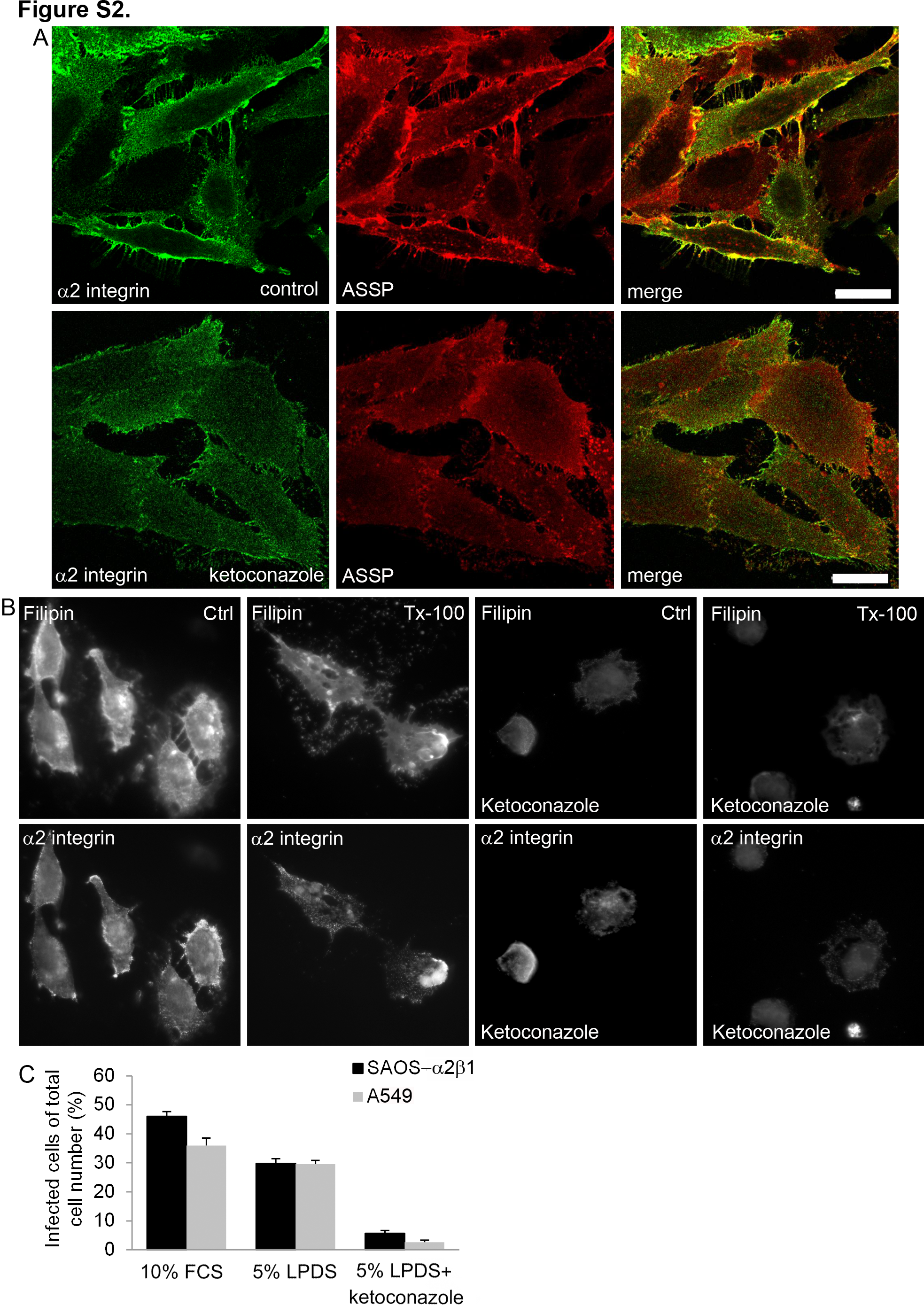

Supplement: Figure S2 — A) Ketoconazole treatment affects the localization of unclustered α2 integrin and aerolysin toxin. SAOS-α2β1 cells pretreated for 3 days ±10 mM ketoconazole were labeled with mouse α2 integrin antibody (A211E10) on ice and after 4% PFA fixation with goat anti-mouse Alexa 488 (green) and Alexa 546-conjugated aerolysin toxin (ASSP, red, Fivaz et al. Embo J. 21∶3989–4000, 2002). Bars 20 µm. B) Ketoconazole treatment affects the localization of clustered α2 integrin and cholesterol on the plasma membrane. SAOS-α2β1 cells pretreated for 3 days ±10 mM ketoconazole were clustered with mouse α2 integrin antibody (A211E10) and goat anti-mouse Alexa 488 on ice. After integrin clustering cells were treated ±0.2% Triton X-100 for 30 min on ice followed by 4% PFA fixation. 0.5 mg/ml filipin was used to label cholesterol at +37°C for 30 min after fixation. C) Ketoconazole treatment inhibits EV1 infection. The effect of 5% LPDS and ketoconazole treatment was tested in human lung carcinoma cell line, A549 and SAOS α2β1 cells. Cells were pretreated with 10% FCS DMEM, 5% LPDS DMEM and 10 mM ketoconazole for 3 days before testing EV1 infectivity. The results are mean values of three independent samples (+ SE). (TIF) [file pone.0055465.s002.tif]

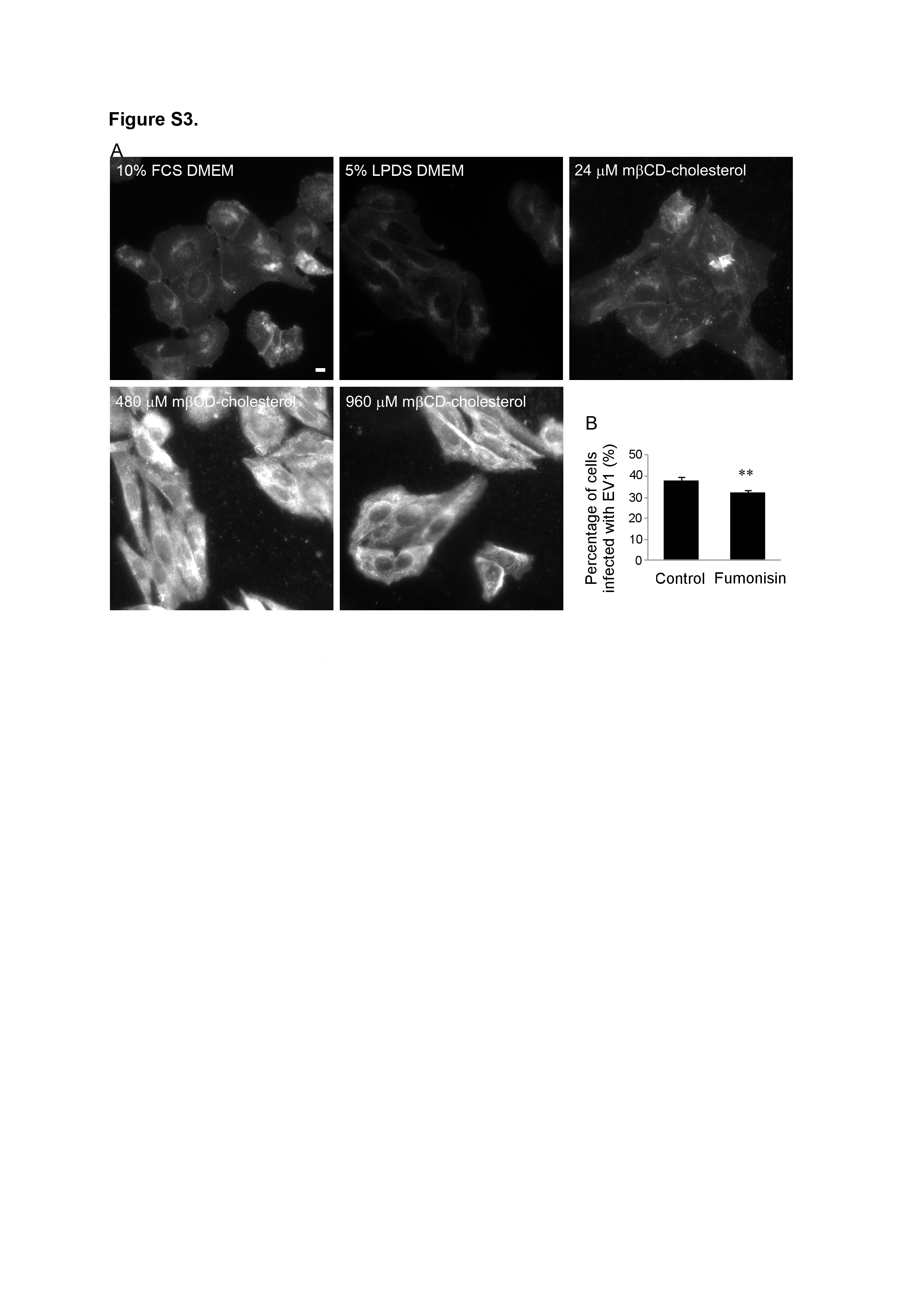

Supplement: Figure S3 — A) mβCD-cholesterol treatment overloads cells with cholesterol. Representative images of cholesterol loading. SAOS-α2β1 cells were grown for 1 day in 5% LPDS before mβCD-chol treatment for 30 min. 4% PFA fixed cells were labeled with 0.5 mg/ml filipin in PBS for 30 min at +37°C. Images were taken with identical wide-field microscope settings. Bar 10 mm. B) Inhibition of sphingomyelin synthesis suppresses EV1 infection. Cells were pretreated with 20 mg/ml Fumonisin B1 in 10% FCS DMEM for 2 days. Drug was also present in infection medium. The results are averages of two independent tests (+SE), more than 1000 cells were counted. (TIF) [file pone.0055465.s003.tif]

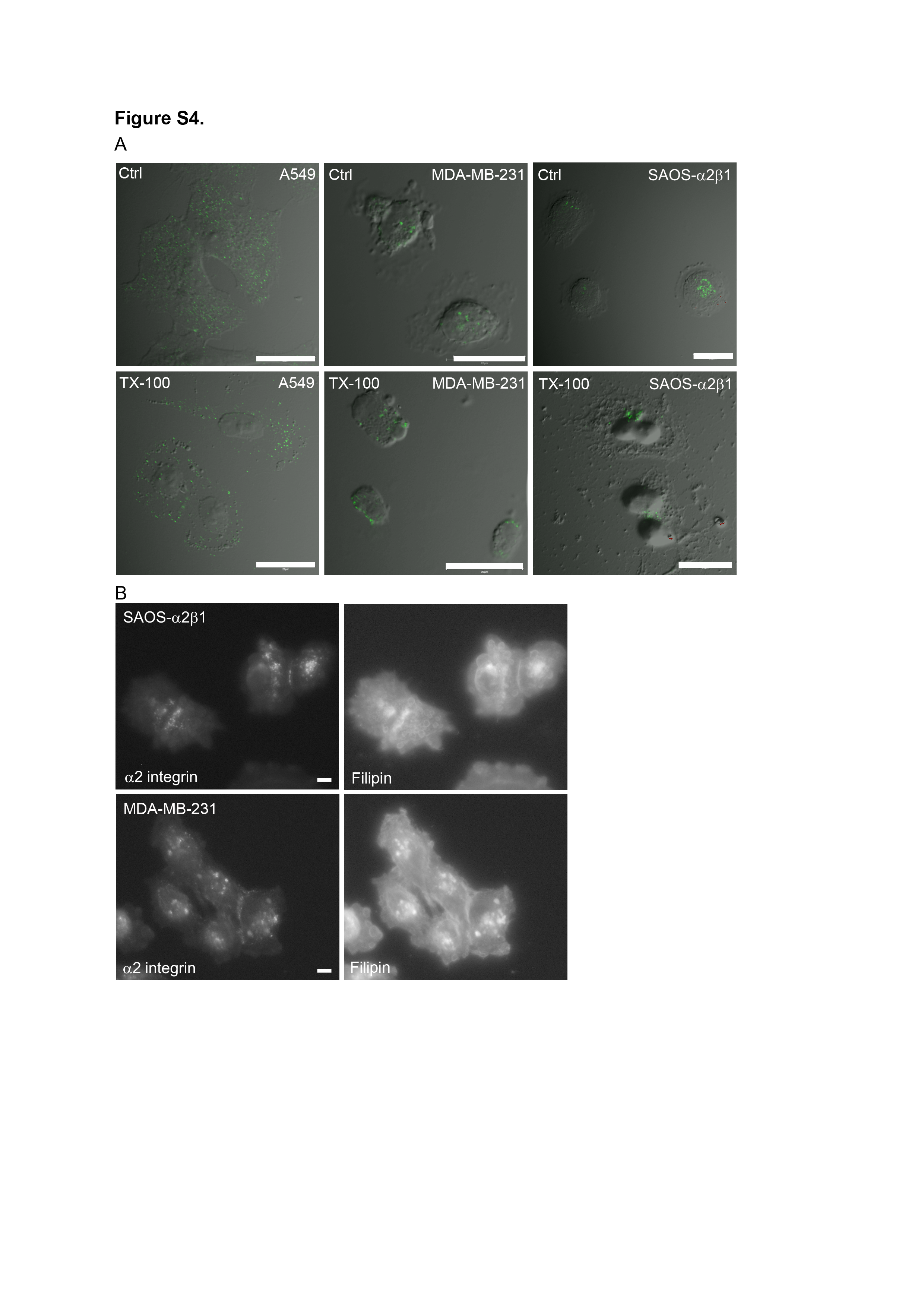

Supplement: Figure S4 — A) α2-MVBs are resistant for cold Triton X-100 treatment in different cell lines. SAOS-α2β1, MDA-MB-231 and A549 cells were clustered with α2 integrin (A211E10) and goat anti-mouse Alexa 488 antibodies on ice. After internalization for 2 hours, cells were treated ±0.2% Triton X-100 for 30 min at +4°C before fixation with 4% PFA. Bars 20 µm. B) Filipin colocalizes with α2 integrin structures. SAOS-α2β1 cells cells were treated with EV1 for 45 min prior α2 integrin was further clustered with sequential antibody treatments on ice (A211E10 and goat anti-mouse Alexa 488, respectively). Virus and integrin were allowed to internalize for 2 h after which cells were fixed with 4% PFA. Cellular cholesterol was labeled after fixation with 0.5 mg/ml filipin in PBS for 30 min at +37°C. Bars 10 µm. (TIF) [file pone.0055465.s004.tif]
